# The Hospital for Diseases of the Skin, Blackfriars

**Published:** 1909-11-13

**Authors:** 


### THE HOSPITAL FOR DISEASES OF THE SKIN, BLACKFRIARS.

### THE ECHOES OF AN OLD DISPUTE.

(An account of the former state of affairs which led to this action will be found in The Hospital for April 20, 1907, volume 42, p. 75.)

The affairs of this hospital, to which allusion has been made in these columns in our issue of April 20, 1907, came before Mr. Justice Warrington in the High Court of Chancery on the 5th and 8th of the present month, in an action in which Colonel Marshall and Messrs. Cutler and Sebastien, the present trustees of the hospital funds, were plaintiffs, and the present Committee of Management the defendants, the Attorney-General being also made a party. It appeared that rules had been made for the management of the hospital which provided, *inter alia*, that subscribers of one guinea upwards (when their subscriptions should amount to £3 3s.) were eligible as governors, so long as their subscriptions continued; that donors of twenty guineas were eligible as life governors; and that, at the next subsequent meeting of the committee, both governors and life governors were to be proposed for election. The annual meeting of governors was to elect the committee of management, which was to consist of governors annually elected and of certain of the medical staff and honorary lay officers. The sole management of the hospital was to be vested in the committee, subject only to control by the annual meeting of governors. It was alleged that no members of the committee in or since 1899 had, in fact, been elected in accordance with the rules, but that they had been elected at meetings of governors and subscribers. In 1899 the committee appointed as trustees two of the plaintiffs, and in 1901 the third plaintiff. At the time of these appointments deeds poll were executed by the trustees, under which they were declared to hold the hospital property in trust for the hospital on the trusts of an earlier deed of 1886. This deed subjected the trustees to the control of the committee, or a majority of the committee, in whom the power of appointing new trustees was vested. The property held by the trustees at the present time amounted to more than £7,000. Doubts had arisen as to the validity of the trustees' appointment, and as to whether the committee had been properly elected. The trustees accordingly brought an action, asking that it should be determined whether they had been duly appointed, and, if so, that they might be discharged; that, if they were not duly appointed, such directions might be given as would enable them properly to discharge themselves; and that the trusts might be executed by the Court.

The defendants (other than the Attorney-General) did not admit that they were not properly elected, and submitted to the directions of the Court. Very little evidence was called by the plaintiffs, and the disputes which have arisen in connection with the hospital affairs were not gone into at any length, being treated as irrelevant in so far as the legal position was concerned, though it was stated, on the defendants' behalf, that they were prepared to meet all charges that had been made against them.

The learned judge, in giving judgment, stated that, in his opinion, the proceedings were properly brought by the plaintiffs, who had found themselves in a difficult position. He refused to accept any suggestion of misconduct on the part of the committee, and stated that, though doubts on the subject existed, it had not been proved that the committee had not been properly elected, and that there was no evidence that for the last two years, at any rate, the committee had not conducted the affairs of the hospital in the best interests of the hospital. Accordingly, in directing that a scheme should be prepared for future management, he gave both parties their costs. He further said that in the interest of the hospital funds he could only permit one of the rival parties among the subscribers to attend in Chambers when the scheme was being settled, and that he must give the preference to those who had had the practical government of the hospital during the past two years, rather than to those who had merely had the custody of its funds; but that the latter could make such representations as they thought fit through the Attorney-General.

This seems to be a happy ending to the disputes which have for so long vexed the hospital and militated against its best interests. We sincerely trust that, with the conclusion of this case, all parties will agree in burying the past, and in uniting to secure in every possible way efficiency, harmony, and sound management in the future.

